# Supplementary material for: Epithelial to mesenchymal transition and M2 macrophages drive aggressiveness in murine and human sarcomatoid urothelial carcinoma
Source: bioRxiv. 2026 Oct 1:2026.09.30.755727. Preprint. [Version 1] doi: 10.64898/2026.09.30.755727 (PMC13648735; doi:10.64898/2026.09.30.755727)

bioRxiv preprint doi: https://doi.org/10.64898/2026.09.30.755727; this version posted October 2, 2026. The copyright holder for this preprint (which was not certified by peer review) is the author/funder, who has granted bioRxiv a license to display the preprint in perpetuity. It is made available under a CC-BY 4.0 International license.

Fig. 1 Supplemental

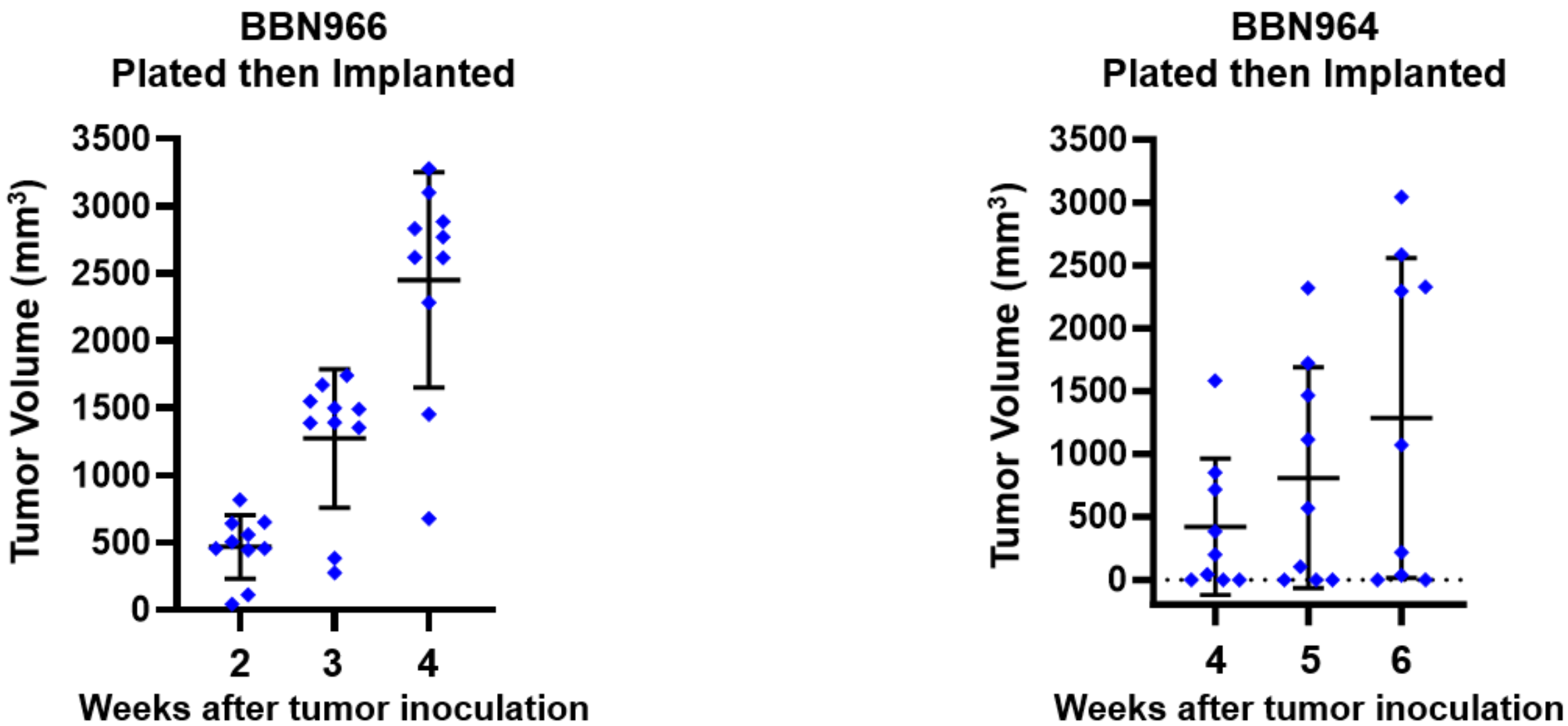

bioRxiv preprint doi: https://doi.org/10.64898/2026.09.30.755727; this version posted October 2, 2026. The copyright holder for this preprint (which was not certified by peer review) is the author/funder, who has granted bioRxiv a license to display the preprint in perpetuity. It is made available under a CC-BY 4.0 International license.

Sup. Fig. 2

GBCI24_38 BBN964	
	**Diagnosis**
**Pathologist #1**	Sarcomatoid carcinoma
**Pathologist #2**	Sarcomatoid carcinoma
**Pathologist #3**	Squamous carcinoma

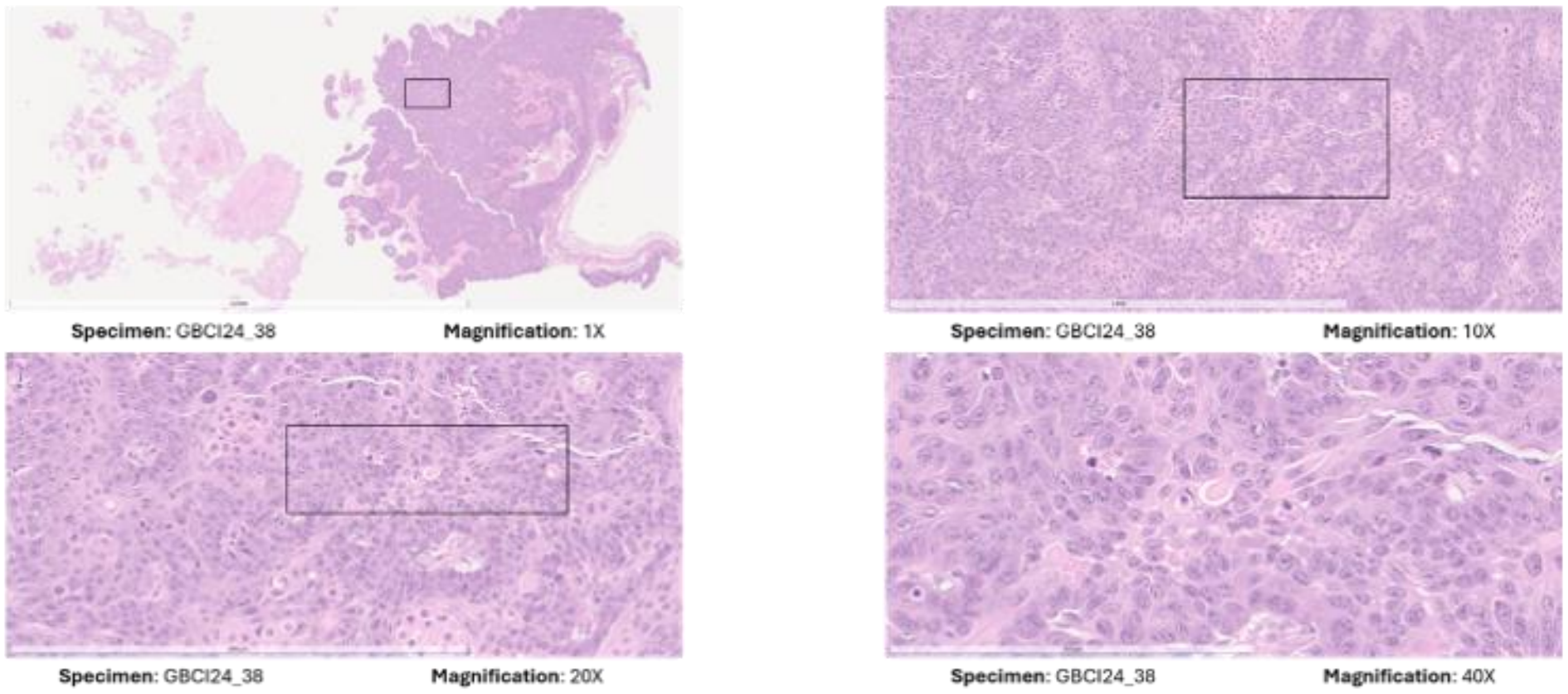

GBCI23_07 BBN966	
	**Diagnosis**
**Pathologist #1**	Sarcomatoid carcinoma with Squamous Features
**Pathologist #2**	Sarcomatoid carcinoma
**Pathologist #3**	Sarcomatoid carcinoma

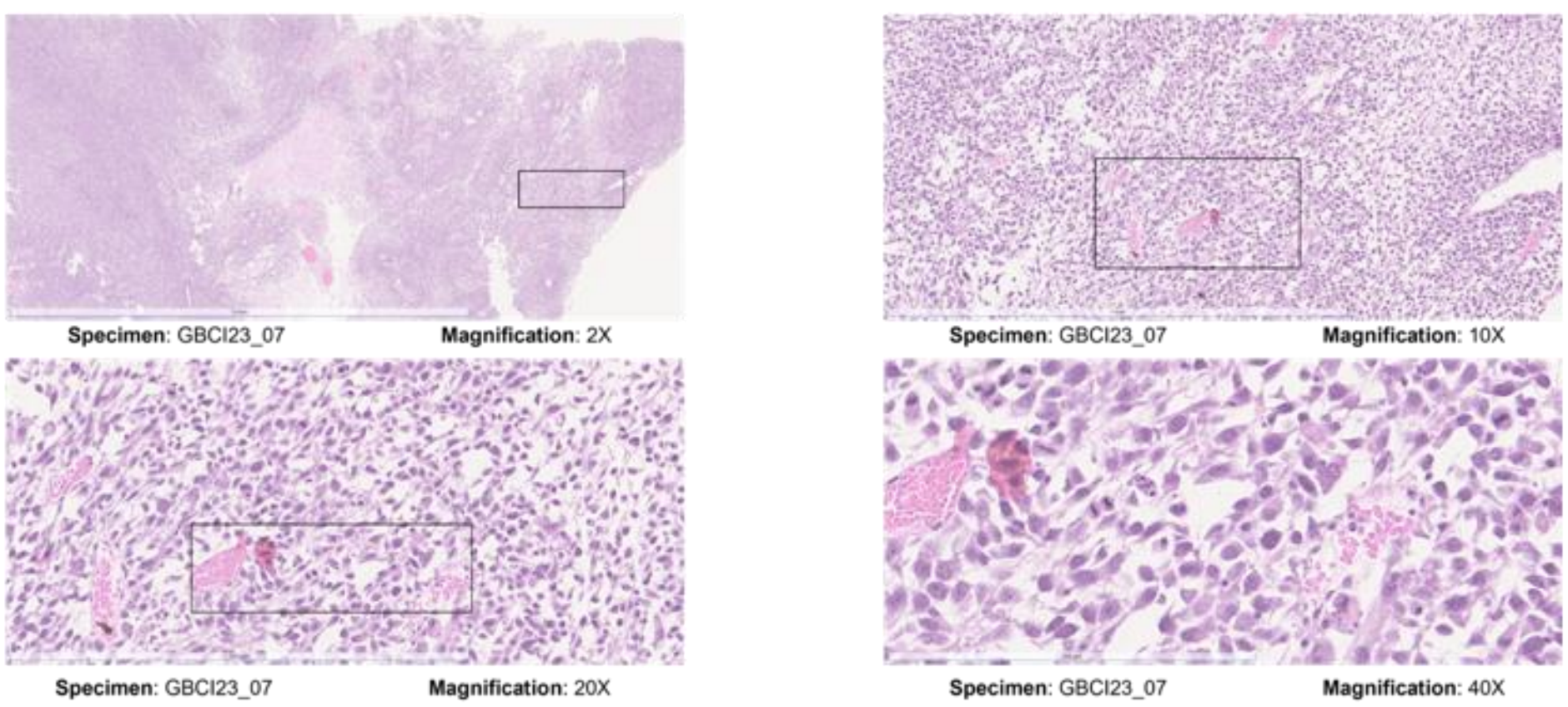

GBCI23_03 BBN975	
	**Diagnosis**
**Pathologist #1**	Sarcomatoid carcinoma with Squamous Features
**Pathologist #2**	Squamous carcinoma
**Pathologist #3**	Squamous carcinoma

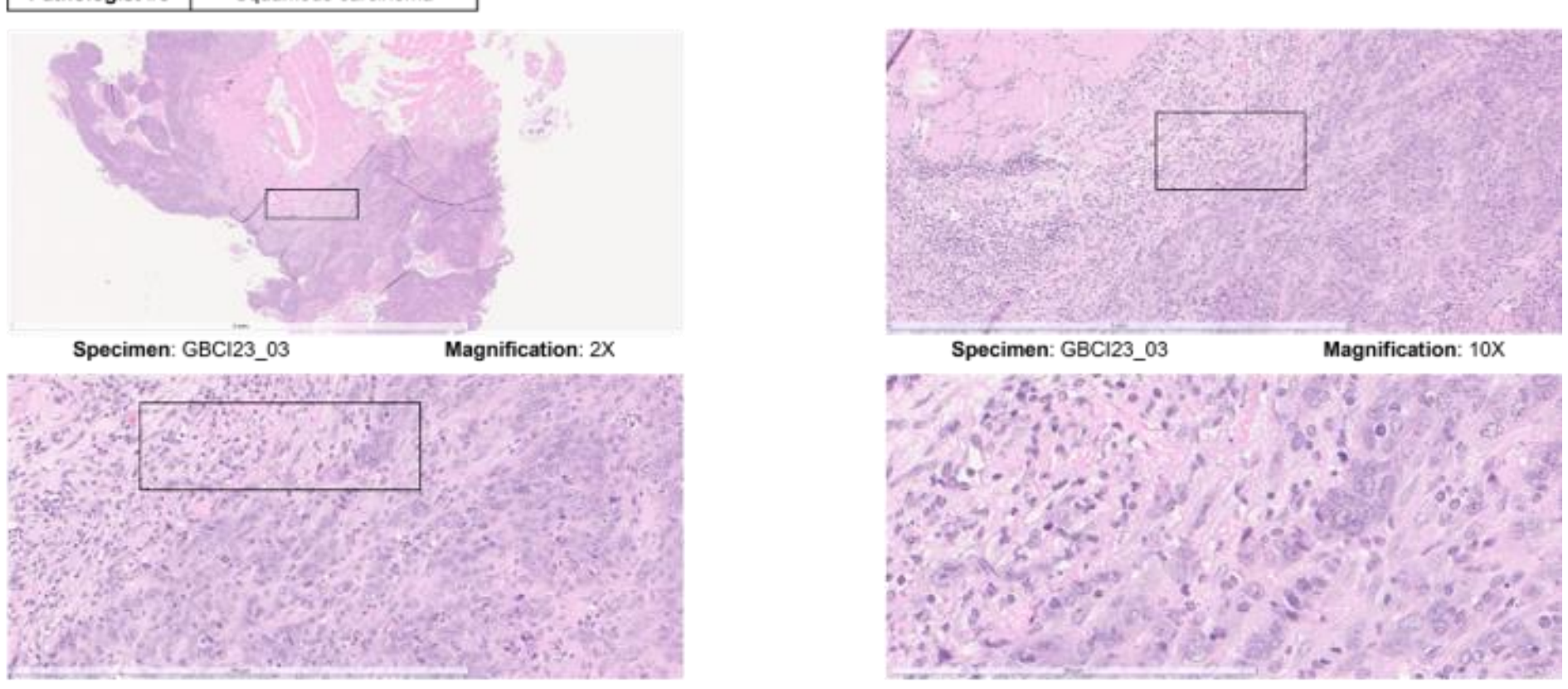

bioRxiv preprint doi: https://doi.org/10.64898/2026.09.30.755727; this version posted October 2, 2026. The copyright holder for this preprint (which was not certified by peer review) is the author/funder, who has granted bioRxiv a license to display the preprint in perpetuity. It is made available under a CC-BY 4.0 International license.

Sup. Table 1

BBN964 Initial Rag1-KO Injection					
Pathologist #1		Pathologist #2		Pathologist #3	
Diagnosis	Frequency	Diagnosis	Frequency	Diagnosis	Frequency
Sarcomatoid carcinoma	12	Squamous carcinoma	10	Squamous carcinoma	14
High grade carcinoma with necrosis	1	Sarcomatoid carcinoma	3		
High grade carcinoma with squamous features	1	Not scanned	1		
**BBN966 Initial Rag1-KO Injection**					
Pathologist #1		Pathologist #2		Pathologist #3	
Diagnosis	Frequency	Diagnosis	Frequency	Diagnosis	Frequency
Sarcomatoid carcinoma	12	Sarcomatoid carcinoma	10	Sarcomatoid carcinoma, Ugly tumor with spindled areas.	1
Invasive high grade with possible sarcomatoid features	1	Sarcomatoid carcinoma and Squamous carcinoma	7	Squamous carcinoma, sarcomatoid carcinoma. 70% squamous, 30% sarcomatoid.	2
Invasive high grade papillary urothelial carcinoma with sarcomatoid features	1			Sarcomatoid carcinoma	10
Sarcomatoid carcinoma arising in papillary urothelial with squamous features	3			Squamous carcinoma, sarcomatoid carcinoma. 10% squamous, 90% sarcomatoid	1
				Squamous carcinoma, sarcomatoid carcinoma. 5% squamous, 95% sarcomatoid	1
				Squamous carcinoma, sarcomatoid carcinoma	1
				Squamous carcinoma, sarcomatoid carcinoma. 40% squamous, 60% sarcomatoid	1
**BBN975 Initial Rag1-KO Injection**					
Pathologist #1		Pathologist #2		Pathologist #3	
Diagnosis	Frequency	Diagnosis	Frequency	Diagnosis	Frequency
Sarcomatoid carcinoma arising in papillary urothelial with squamous features	4	Squamous carcinoma	16	Squamous carcinoma	14
Sarcomatoid carcinoma	1	Sarcomatoid carcinoma and squamous carcinoma	1	Squamous carcinoma, sarcomatoid carcinoma. 30% squamous, 70% sarcomatoid	3
High grade carcinoma	1	Not scanned	1	Not Scanned	1
Sarcomatoid carcinoma with squamous features	12				

bioRxiv preprint doi: https://doi.org/10.64898/2026.09.30.755727; this version posted October 2, 2026. The copyright holder for this preprint (which was not certified by peer review) is the author/funder, who has granted bioRxiv a license to display the preprint in perpetuity. It is made available under a CC-BY 4.0 International license.

Sup. Table 2-3

BBN964 Rag1-KO to Rag1-KO Recycled					
Pathologist #1		Pathologist #2		Pathologist #3	
Diagnosis	Frequency	Diagnosis	Frequency	Diagnosis	Frequency
Sarcomatoid carcinoma	10	Sarcomatoid carcinoma	10	Squamous carcinoma	10
**BBN966 Rag1-KO to Rag1-KO Recycled**					
Pathologist #1		Pathologist #2		Pathologist #3	
Diagnosis	Frequency	Diagnosis	Frequency	Diagnosis	Frequency
Sarcomatoid carcinoma	16	Sarcomatoid carcinoma	17	Sarcomatoid carcinoma	15
High grade carcinoma	1			Squamous carcinoma, sarcomatoid carcinoma, <5% squamous, remaining is sarcomatoid	2
**BBN975 Rag1-KO to Rag1-KO Recycled**					
Pathologist #1		Pathologist #2		Pathologist #3	
Diagnosis	Frequency	Diagnosis	Frequency	Diagnosis	Frequency
Sarcomatoid carcinoma with squamous features	15	Squamous carcinoma	15	Squamous carcinoma	15

BBN964 Rag1-KO to Rag1-KO to B6 Recycled					
Pathologist #1		Pathologist #2		Pathologist #3	
Diagnosis	Frequency	Diagnosis	Frequency	Diagnosis	Frequency
Sarcomatoid carcinoma	3	Sarcomatoid carcinoma	3	Squamous carcinoma	3
**BBN966 Rag1-KO to Rag1-KO to B6 Recycled**					
Pathologist #1		Pathologist #2		Pathologist #3	
Diagnosis	Frequency	Diagnosis	Frequency	Diagnosis	Frequency
Sarcomatoid carcinoma with squamous features	1	Sarcomatoid carcinoma	5	Sarcomatoid carcinoma	5
Sarcomatoid carcinoma	4				
**BBN975 Rag1-KO to B6 Recycled**					
Pathologist #1		Pathologist #2		Pathologist #3	
Diagnosis	Frequency	Diagnosis	Frequency	Diagnosis	Frequency
High grade carcinoma	1	Squamous carcinoma	6	Squamous carcinoma	6
Sarcomatoid carcinoma with squamous features	5				

bioRxiv preprint doi: https://doi.org/10.64898/2026.09.30.755727; this version posted October 2, 2026. The copyright holder for this preprint (which was not certified by peer review) is the author/funder, who has granted bioRxiv a license to display the preprint in perpetuity. It is made available under a CC-BY 4.0 International license.

Supplemental Fig. 3

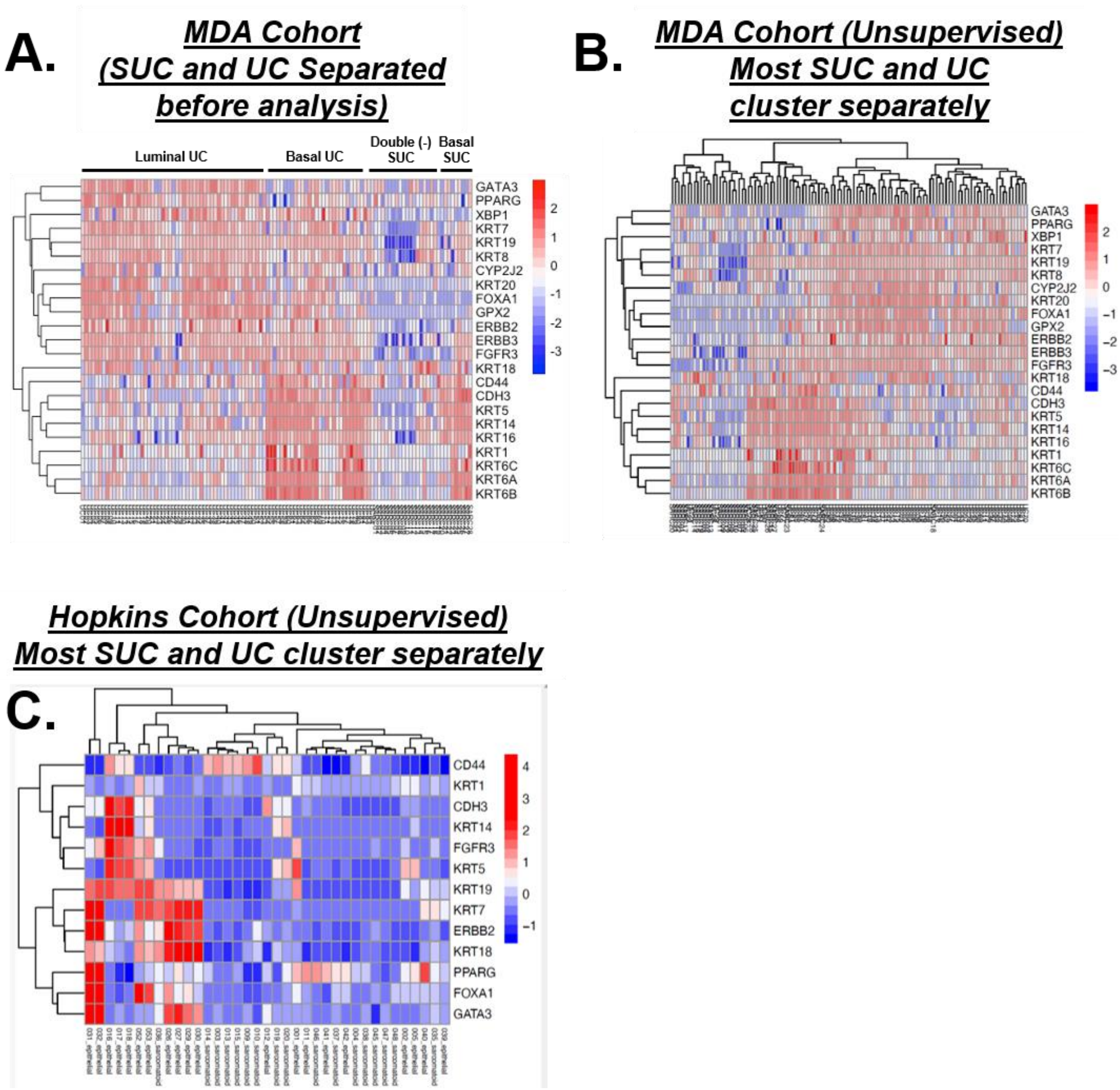

bioRxiv preprint doi: https://doi.org/10.64898/2026.09.30.755727; this version posted October 2, 2026. The copyright holder for this preprint (which was not certified by peer review) is the author/funder, who has granted bioRxiv a license to display the preprint in perpetuity. It is made available under a CC-BY 4.0 International license.

Sup. Fig. 4

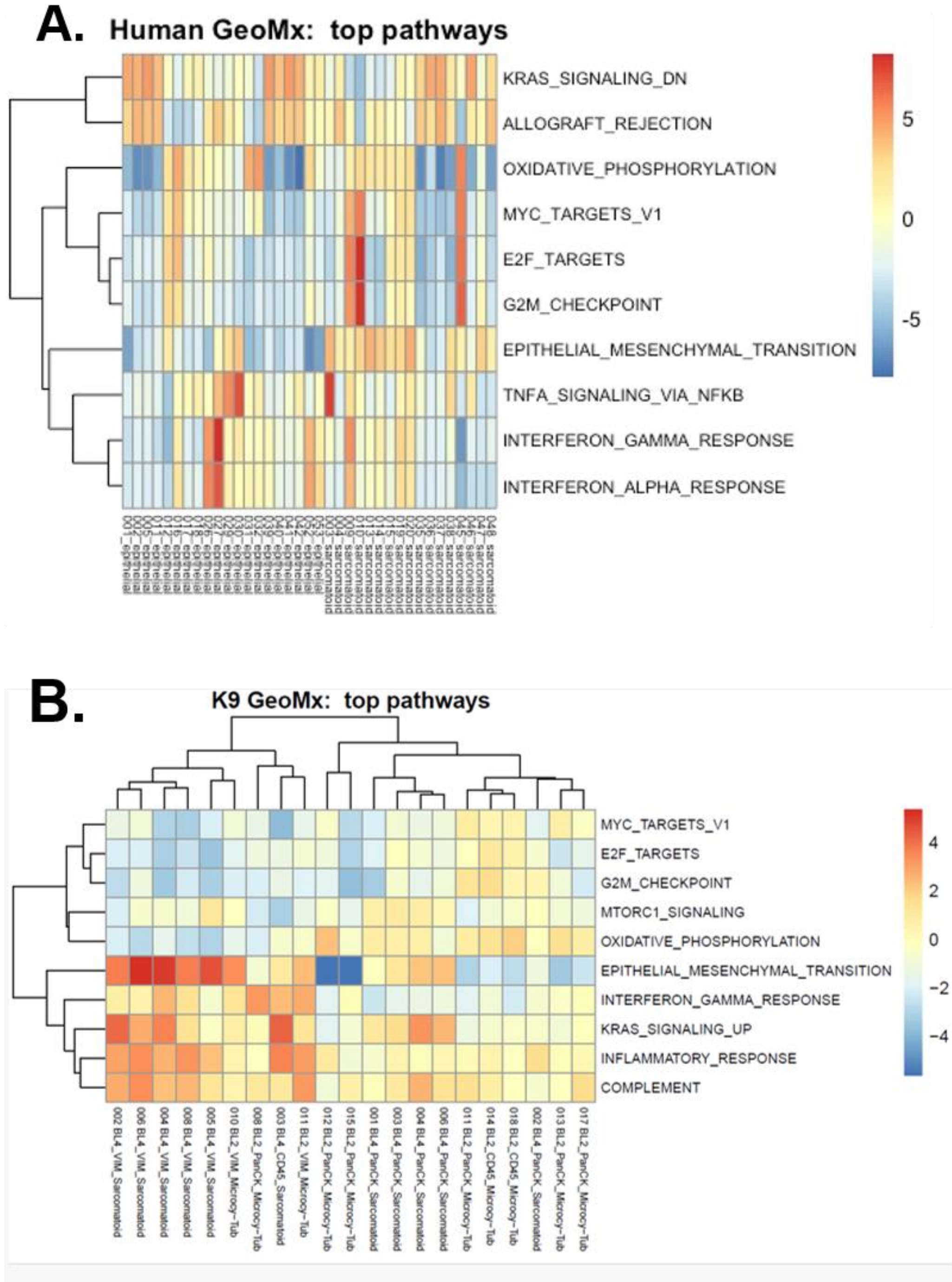

bioRxiv preprint doi: https://doi.org/10.64898/2026.09.30.755727; this version posted October 2, 2026. The copyright holder for this preprint (which was not certified by peer review) is the author/funder, who has granted bioRxiv a license to display the preprint in perpetuity. It is made available under a CC-BY 4.0 International license.

Sup. Fig. 5

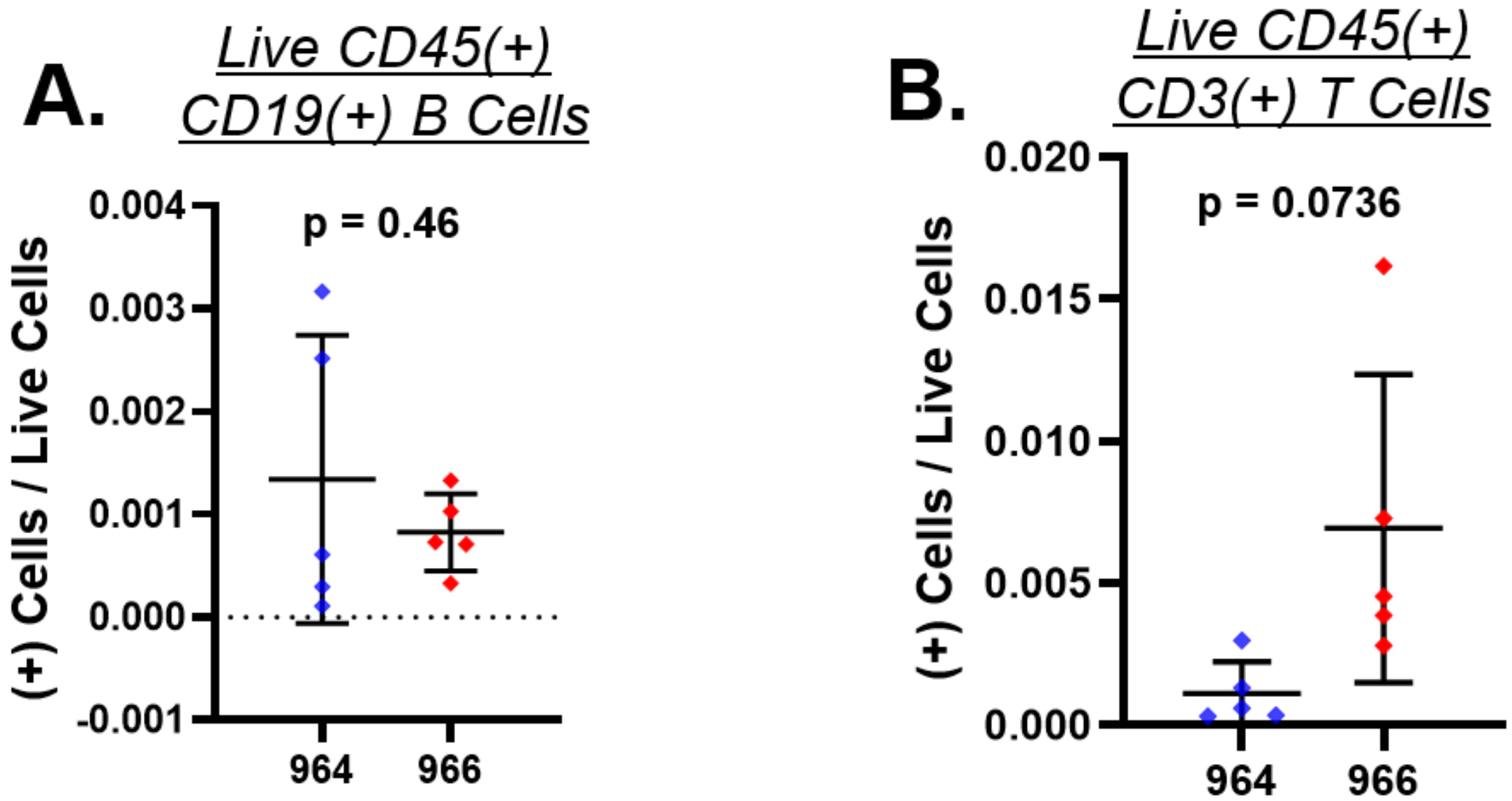

Supplement: Supplementary file 1 [file NIHPP2026.09.30.755727V1-supplement-1.pdf]
